# On the role of body size, brain size, and eye size in visual acuity

**DOI:** 10.1007/s00265-017-2408-z

**Published:** 2017-11-29

**Authors:** Alberto Corral-López, Maddi Garate-Olaizola, Severine D. Buechel, Niclas Kolm, Alexander Kotrschal

**Affiliations:** Department of Zoology/Ethology, Stockholm University, Svante Arrhenius väg 18B, SE-10691 Stockholm, Sweden

**Keywords:** Sensory system, Eye size, Optomotor response, Guppies, Sex differences, Body size

## Abstract

**Abstract:**

The visual system is highly variable across species, and such variability is a key factor influencing animal behavior. Variation in the visual system, for instance, can influence the outcome of learning tasks when visual stimuli are used. We illustrate this issue in guppies (*Poecilia reticulata*) artificially selected for large and small relative brain size with pronounced behavioral differences in learning experiments and mate choice tests. We performed a study of the visual system by quantifying eye size and optomotor response of large-brained and small-brained guppies. This represents the first experimental test of the link between brain size evolution and visual acuity. We found that female guppies have larger eyes than male guppies, both in absolute terms and in relation to their body size. Likewise, individuals selected for larger brains had slightly larger eyes but not better visual acuity than small-brained guppies. However, body size was positively associated with visual acuity. We discuss our findings in relation to previous macroevolutionary studies on the evolution of brain morphology, eye morphology, visual acuity, and ecological variables, while stressing the importance of accounting for sensory abilities in behavioral studies.

**Significance statement:**

Pre-existing perceptual biases can be keys for the development of specific behavioral patterns. Hence, potential differences in sensory systems need to be taken into account in the study of animal behavior. We highlight this necessity concentrating on the visual domain and using experimental data on brain size-selected guppies in which we assessed eye size and visual acuity. Behavioral differences between large-brained and small-brained guppies in learning and mate choice predominantly relied on tests using visual cues. Analyses of visual capabilities in this system are therefore necessary. Furthermore, this system offers the unprecedented opportunity to experimentally test the relationship between brain size, eye morphology, and visual capabilities. Our results show similar visual acuities between large-brained and small-brained guppies. However, the differences observed in eye area between the sexes, together with the observed positive relationship between body size and visual acuity, highlight the need to incorporate perceptive differences in the study of animal behavior.

**Electronic supplementary material:**

The online version of this article (10.1007/s00265-017-2408-z) contains supplementary material, which is available to authorized users.

## Introduction

Sensory systems provide the interface between organisms and their environment and are hence crucial for an individual’s fitness. The visual system is particularly important for a wide range of species, including humans, when foraging, selecting a mate, or escaping from potential predators. In such species, visual abilities should be strongly influenced by natural selection. Yet, visual abilities vary markedly across species in the animal kingdom. For instance, visual acuity, the ability of an individual to discriminate spatial detail (Land and Nilsson 2012), varies dramatically even within the closely related species of poeciliid fishes (Douglas and Hawryshyn 1990).

Differences in ecological requirements form one important factor shaping visual evolution across species (von der Emde and Warrant 2016). In particular, comparative analyses suggest that visual challenges related to type of diet, diel activity patterns, or habitat complexity drive the evolution of visual acuity (Hutcheon et al. 2002; Veilleux and Kirk 2014; Caves et al. 2017). The retina develops as an outpouching of the lateral neural tube (Kuzawa et al. 2014) and is therefore considered to form a part of the brain. As brain tissue is among the most energetically costly tissues in the vertebrate body (reviewed in Niven 2016), large eyes should be costly to evolve and energetic demands may therefore constrain the evolution of visual acuity (Niven and Laughlin 2008). Apart from overall eye size, there are several other proximate aspects of the visual system that are associated with visual acuity. For instance, eye length is positively correlated with visual acuity across mammals (Heesy and Hall 2010; Veilleux and Kirk 2014), birds (Hall and Heesy 2011), and ray-finned fishes (Caves et al. 2017). Further, the density of photoreceptors and retinal ganglion cells within the retina are key factors in the variation of visual acuity across species (Douglas and Hawryshyn 1990; Fernández-Juricic 2012). This is so because more photoreceptors in the retina lead to a smaller distance between two adjacent receptors, which increases the ability to focus images (Douglas and Hawryshyn 1990; Haug et al. 2010), and because information from the photoreceptors is collected in retinal ganglion cells, which then carry the information through the optic nerve (Collin et al. 1999). Additionally, visual acuity can depend on the morphology of the regions in the central nervous system where visual information is processed (reviewed in Iwaniuk 2016). Studies across bird species suggest that the size of several neural vision-related regions positively correlates with increased capacity of processing visual information (Wylie et al. 2015). In cartilaginous and cyprinid fishes, species inhabiting areas with higher dependence on vision show a larger optic tectum size (Brandstätter and Kotrschal 1990; Yopak and Lisney 2012).

While the proximate and ultimate factors underlying differences in visual abilities seem well established, to date, such studies linking vision, eye anatomy, and brain anatomy are correlational and do not allow for ascertaining causality. A recent artificial selection experiment on relative brain size in guppies (*Poecilia reticulata*; Kotrschal et al. 2013) offers the opportunity to experimentally test the link between brain morphology and visual acuity. These selection lines that differ by up to 15% in relative brain size do not differ in body size, but show a range of physiological, behavioral, and life history differences (Kotrschal et al. 2013, 2015a, 2016; van der Bijl et al. 2015; Corral-López et al. 2017). As selective pressures can be powerful modulators of the sensory systems, it is important to determine whether these fish exposed to different brain size selection regimes might differ in their sensory abilities. This is particularly important because assessment of cognitive performance of these selection lines in numerical-learning, reversal-learning, and maze-learning assays relied on the presentation of visual stimuli (Kotrschal et al. 2013, 2015b; SDB unpubl. data). Likewise, mate choice preferences were assayed by visually presenting potential partners (Corral-López et al. 2017). Besides the unique opportunity to experimentally test the link between brain morphology and vision, testing visual acuity will hence clarify if potential differences in the visual system may have contributed towards the apparent behavioral differences previously found in these selection lines. In a similar artificial selection experiment for brain weight in mice, some of the founder inbred strains presented retinal degeneration which was fixed with higher frequency in lines selected for smaller brain weight (Roderick et al. 1973). This fact stresses the need for evaluating visual capabilities of individuals selected for morphological differences in their brain anatomy. Therefore, we here quantitatively test whether directional selection for large and small relative brain size impacts eye size and visual acuity.

Fishes show a large variation in visual acuity (Caves et al. 2017) and are therefore excellent model organisms to study the relationship between eye morphology and visual acuity (reviewed in Douglas and Hawryshyn 1990). In particular, behavioral measurements of stereotypical innate optomotor responses towards contrasting gratings have been widely used to assess visual acuity of several fish species (Clark 1981; Neave 1984; Haug et al. 2010). The optomotor response describes the change of movement that can be observed when animals follow the movement of vertical stripes (Anstis et al. 1998). In guppies, measurements of optomotor responses have successfully been used to study different aspects of visual performance, such as wavelength sensitivity (Anstis et al. 1998) in wild-type fish. We have previously used this approach to show that the brain size selection lines perform equally well in color discrimination tasks (Corral-López et al. 2017).

As the eye is developmentally an integrated part of the brain, and in the guppy selection lines, the 11 major brain regions do not differ in relative proportions between large-brained and small-brained animals (Kotrschal et al. 2017), we predict absolutely larger eyes in the large-brained animals as compared to the small-brained animals. Larger eyes typically confer better vision in fishes (Caves et al. 2017); we may hence expect higher visual acuity of large-brained fish. This detailed test of visual acuity will elucidate whether, and if so to what extent, the brain size lines differ in visual acuity and whether this difference is large enough to have impacted the results of previous behavioral assays. Additionally, female guppies are substantially larger than male guppies, with larger brains and eyes; we therefore predict a higher visual acuity in females compared to males.

## Methods

### Study system

To experimentally test the effect of brain size evolution on eye size and visual acuity, we used male and female guppies artificially selected for relative brain size. A detailed description of how the artificial selection procedure was undertaken can be found in Kotrschal et al. (2013). In summary, parental brain weight data corrected for body size was used for artificial selection during four generations to generate three lines with large relative brain size and three lines with small relative brain size. These replicate selection lines showed a 13.6% difference in relative brain size in the fourth generation (Kotrschal et al. 2015b). After this generation, 30 non-related males and females from each line were paired (180 pairs) to generate a fifth generation of selected offspring. All fish were removed from their parental tanks after birth, separated by sex at the first onset of sexual maturation, and then kept in groups of 10 individuals in 7-l tanks containing java moss, 2 cm of gravel, and biological filters. We allowed visual contact between these tanks. These animals differed by approximately 12.5% (AK unpubl. data) in relative brain size. Prior to the behavioral trials and morphological measurements, all fish were individually isolated in 2-l tanks. To avoid isolation stress, we allowed for visual contact between the tanks. The laboratory was maintained at 26 °C with a 12:12 light/dark schedule. Fish were fed alternating flake food and freshly hatched brine shrimp 6 days per week.

### Morphological analyses

We measured eye size in 30 male and 30 female adult guppies of similar age (approximately 9 months old), balanced for brain size and replicate. Fish were anesthetized with benzocaine (40 mg/l), and pictures from the left side were taken with a Canon EOS 1200. Pictures were analyzed in ImageJ (Schneider et al. 2012) to collect information on eye area and standard body area. Eye and body area was measured by tracing the boundary of the eye and the whole body excluding the fins. Using these two variables, we calculated the ratio between eye area and body area (relative eye area) for every fish. Image analyses were performed blind to the treatment as running numbers were uninformative of experimental treatment.

### Visual acuity analyses

In another cohort of animals, we assessed the effect of brain size selection on the visual resolution of female and male guppies by determining the smallest stimulus size, which elicited an optomotor response. We quantified the optomotor response of 60 adult guppies: 30 males (15 large-brained versus 15 small-brained) and 30 females (15 large-brained versus 15 small-brained). All fish were fully grown adults of similar age (approximately 9 months old) from the fifth generation of artificial selection. We defined optomotor response as the time that each fish spent circling in the same direction as a clockwise rotational stimulus. We measured this optomotor circling in response to a rotating stimulus (vertical black and white stripes of different widths) that was projected on the walls of a round white arena. For this, we exposed guppies to 12 different rotating stimuli that gradually decrease in the widths of the stripes from 2.40 to 0.20 cm. These widths correspond to theoretical value of visual acuity between 0.84 and 1.49 cycles per degree obtained by calculating the arctan of the angle of the triangle formed by the minimum distance at which the stripes could be observed (10 cm, see below) and the separation between stripes (Douglas and Hawryshyn 1990). As the speed of the moving stripes may impact optomotor response, we used four different speeds at which we presented the stimuli (from 157 to 628 cm/s). The speed at which the stimuli moved did not alter the observed optomotor response (see Supplementary material 1; Fig. S1). After visual inspection of the response towards the whole range of widths presented (Supplementary material 2: Fig. S2), we generated a video recording with the rotating stimuli at equal speed and with widths of stripes at the lower end of guppy visual acuity (0.34 to 0.20-cm widths). Each rotating stimulus lasted 60 s, and the order of the stimuli in the video was random in relation to the widths of the stripes. Moreover, prior to each rotating stimulus, we included a static image of that same stimulus during 60 s. The behavior of the fish during the presentation of static images of the stimuli was used to assess baseline motion of the fish in a circular pattern when no rotating stimulus was presented.

Fish were individually placed in a white circular ring tank with an inner diameter of 25 cm and an outer diameter of 50 cm. In the ring tank, a transparent Perspex ring of 40-cm diameter set the focal fish’s distance to the outer wall of the ring tank to 10 cm (Supplementary material 3: Fig. S3). The order of the test was randomized regarding sex and brain size selection line. After 2 min of acclimation to the experimental arena, the video recording was started and projected on the outer walls of the ring tank using an Infocus IN114 projector (Supplementary material 3: Fig. S3). Behavior of fish was recorded using a Sony Cam HDR-DR11E and later scored by a single observer (MG-O) using BORIS v. 2.72 (Friard and Gamba 2016). We quantified optomotor circling by scoring the time that each fish spent circling clockwise (same direction as the rotational stimulus) and the time that fish spent circling anti-clockwise. Anti-clockwise circling was not included in the statistical analyses as it was generally rare and did not differ between brain size treatments (means ± SE: estimate_large-brained_ 0.06 ± 0.01, estimate_small-brained_ 0.05 ± 0.01; LMM_optomotor response_: brain size: *χ* ^2^ = 0.77, df = 1, *p* = 0.383). Behavioral scoring was performed blind to the treatment since only running numbers identified individuals. Likewise, scoring was blind to the rotation and bandwidth of the stripes since only the fish, but not the rotating stimuli, were visible during scoring. We quantified body size of all individuals by measuring their standard length (from the tip of the snout to the end of the caudal peduncle) to the nearest 0.1 mm using calipers after the test.

### Data analyses

Since eye morphological features are commonly used as proxies of visual acuity (Hughes 1977; Veilleux and Kirk 2014), we used linear mixed-effects models (LMMs) to test for potential differences in the morphological variables between the sexes and between the relative brain size selection lines. We used body size, eye area, and relative eye area as the dependent variable in three independent models. All models included brain size selection regime (large or small) and sex as fixed effects. In addition, models included a random intercept for each replicate selection line and a random slope for brain size within each of the three replicates. Model diagnostics showed that residual distributions were roughly normal with no signs of heteroscedascity.

We evaluated potential differences in visual acuity between the fish by studying their differences in optomotor response. First, to validate the use of this variable as a proxy of visual acuity, we assessed the general response of the fish towards the rotational stimuli independently for every stimulus. For this purpose, we compared the time that every individual spent performing optomotor response during the rotation phase and acclimation phase of the different stimuli presented. As such, we used a LMM with total optomotor response as the dependent variable, rotation of the stimulus as the fixed effect, and the id of the fish as a random factor. Second, to study the effect of sex and brain size selection in visual acuity, we used a LMM with optomotor response as the dependent variable. We used sex and brain size selection regime as fixed effects and fish body size as a covariate. Full models included all interactions between fixed effects and the covariate. In addition, models included a random intercept for each replicate selection line and a random slope for brain size selection regime within each replicate. Stepwise model selection based on Akaike information criterion indicated a better fit of the model without any interaction between fixed effects and the covariate (Supplementary material 4: Table S1). Model diagnostics showed that residual distributions were roughly normal with no signs of heteroscedascity. We further investigated differences in optomotor response between large-brained and small-brained males and females. For this, we used a LMM with the optomotor response for every bandwidth as the dependant variable, brain size selection regime as fixed effects, and body size as a covariate. All models included a random intercept for each replicate selection line and a random slope for brain size selection regime within each replicate. Due to the multiple testing procedure, we applied false discovery rate correction to the 0.05 significance level to reject the null hypothesis of no differences in optomotor response (Benjamini and Hochberg 1995).

Finally, to determine whether an absence of a significant difference was a good indicator of no difference between groups, we performed a power analyses by (i) calculating the observed effect size (Cohen’s *d*) of our LMM (Nakagawa and Cuthill 2007) and (ii) using Monte Carlo simulations of the LMM (*n* = 1000) to calculate the statistical power of our LMM under scenarios of increased sample size using the R package “simr” (Green and MacLeod 2016). All analyses were performed in R v. 3.3.2 (R Core Team 2015).

## Results

### Morphological analyses

**Fig. 1:**
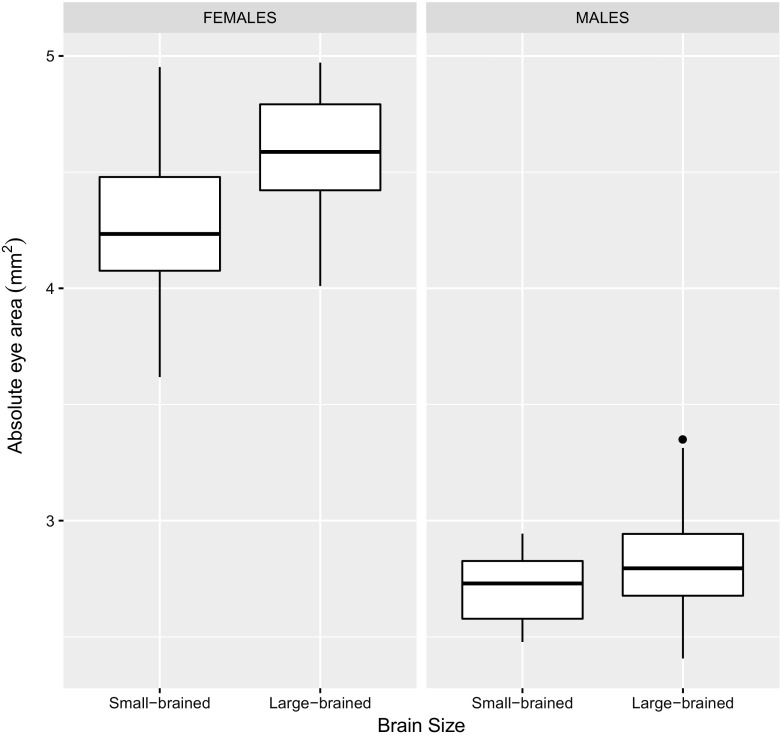
Absolute values of eye size in females and males artificially selected for relative brain size. Large-brained guppies (*n* = 30) had larger eyes than small-brained guppies (*n* = 30) (*p* = 0.025). Likewise, females (*n* = 30), regardless of relative brain size selection treatment, had larger eyes than males (*n* = 30) in this species (*p* < 0.001). Box plots indicate the median, first and third quartiles (hinges), datum within 1.5 IQR of first and third quartiles (whiskers), and outlier points outside the 1.5 IQR

**Fig. 2:**
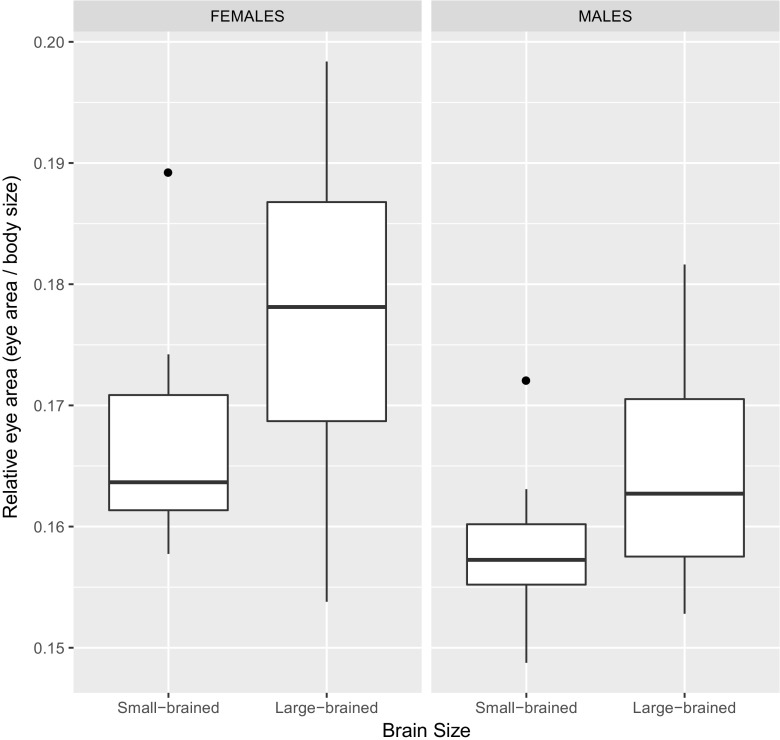
Eye area in relation to body area in females and males artificially selected for relative brain size. Large-brained guppies (*n* = 30) had larger relative eye size than small-brained guppies (*n* = 30) (*p* < 0.001). Likewise, females (*n* = 30), regardless of relative brain size selection treatment, had larger relative eye size than males (*n* = 30) (*p* < 0.001). Box plots indicate the median, first and third quartiles (hinges), datum within 1.5 IQR of first and third quartiles (whiskers), and outlier points outside the 1.5 IQR

Large-brained individuals had a larger absolute eye area than small-brained individuals (means ± SE: estimate_large-brained_ 3.72 ± 0.05 mm^2^, estimate_small-brained_ 3.48 ± 0.05 mm^2^; LMM_eye area_: brain size: *χ* ^2^ = 11.61, df = 1, *p* = 0.025; Fig. 1). Such difference in eye area could not be attributed to differences in body size between the brain size selection lines (means ± SE: estimate_large-brained_ 21.60 ± 0.42 mm^2^, estimate_small-brained_ 21.35 ± 0.42 mm^2^; LMM_body size_: brain size: *χ* ^2^ = 0.18, df = 1, *p* = 0.693). Indeed, analyses of relative eye area revealed that large-brained individuals had larger eyes in relation to their bodies than small-brained ones (means ± SE: estimate_large-brained_ 0.171 ± 0.001, estimate_small-brained_ 0.162 ± 0.001; LMM_relative eye area_: brain size: *χ* ^2^ = 14.29, df = 1, *p* < 0.001; Fig. 2).

Females had significantly larger eyes than males, both in absolute terms (means ± SE: estimate_females_ 4.42 ± 0.05 mm^2^, estimate_males_ 2.77 ± 0.05 mm^2^; LMM_eye area_: sex: *χ* ^2^ = 544.97, df = 1, *p* < 0.001; Fig. 1) and in relative terms, when controlling for body size (means ± SE: estimate_females_ 0.172 ± 0.001, estimate_males_ 0.161 ± 0.001; LMM_relative eye area_: sex: *χ* ^2^ = 21.53, df = 1, *p* < 0.001; Fig. 2).

### Visual acuity analyses

**Fig. 3:**
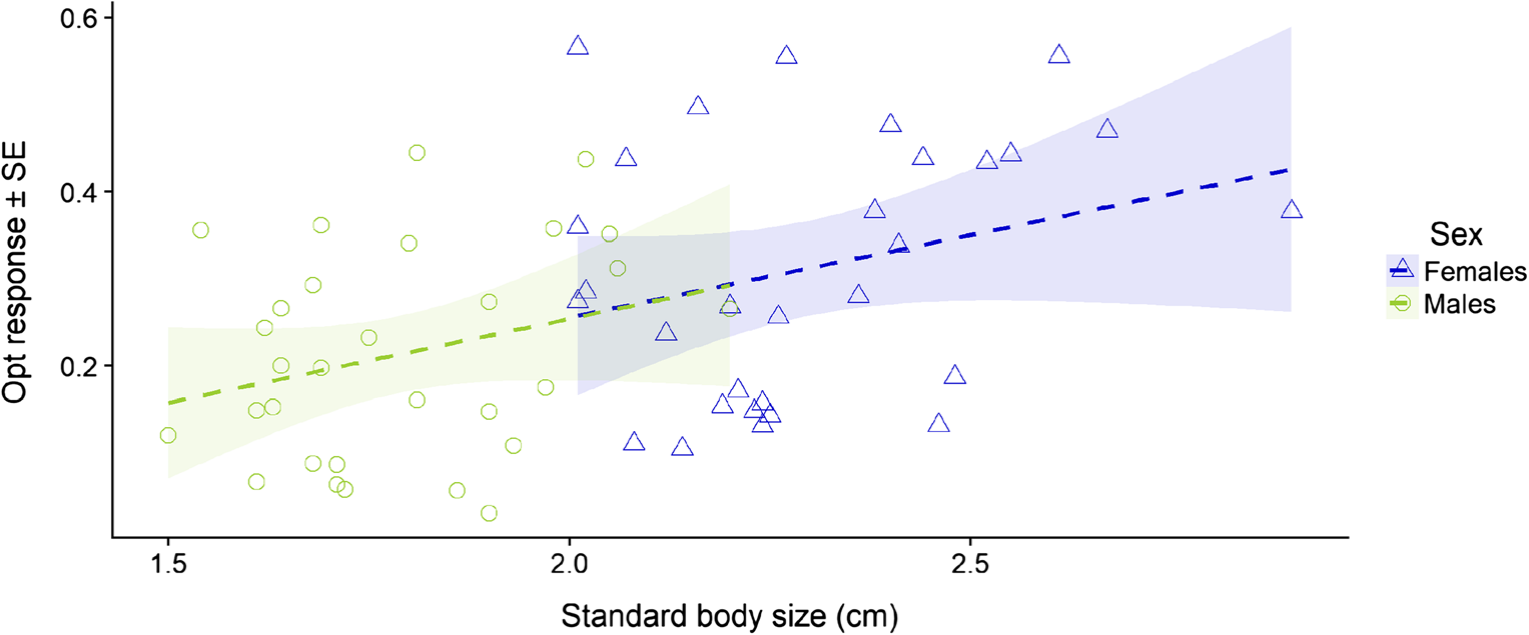
Relationship between visual acuity and body size. Measurements in male and female guppies artificially selected for relative brain size (*n* = 60) indicated a positive correlation between the optomotor response towards a rotational stimuli and the body size of the individual (*p* = 0.036). Linear regression slopes of male (green) and female (blue) values were not significantly different (*p* = 0.957)

**Fig. 4:**
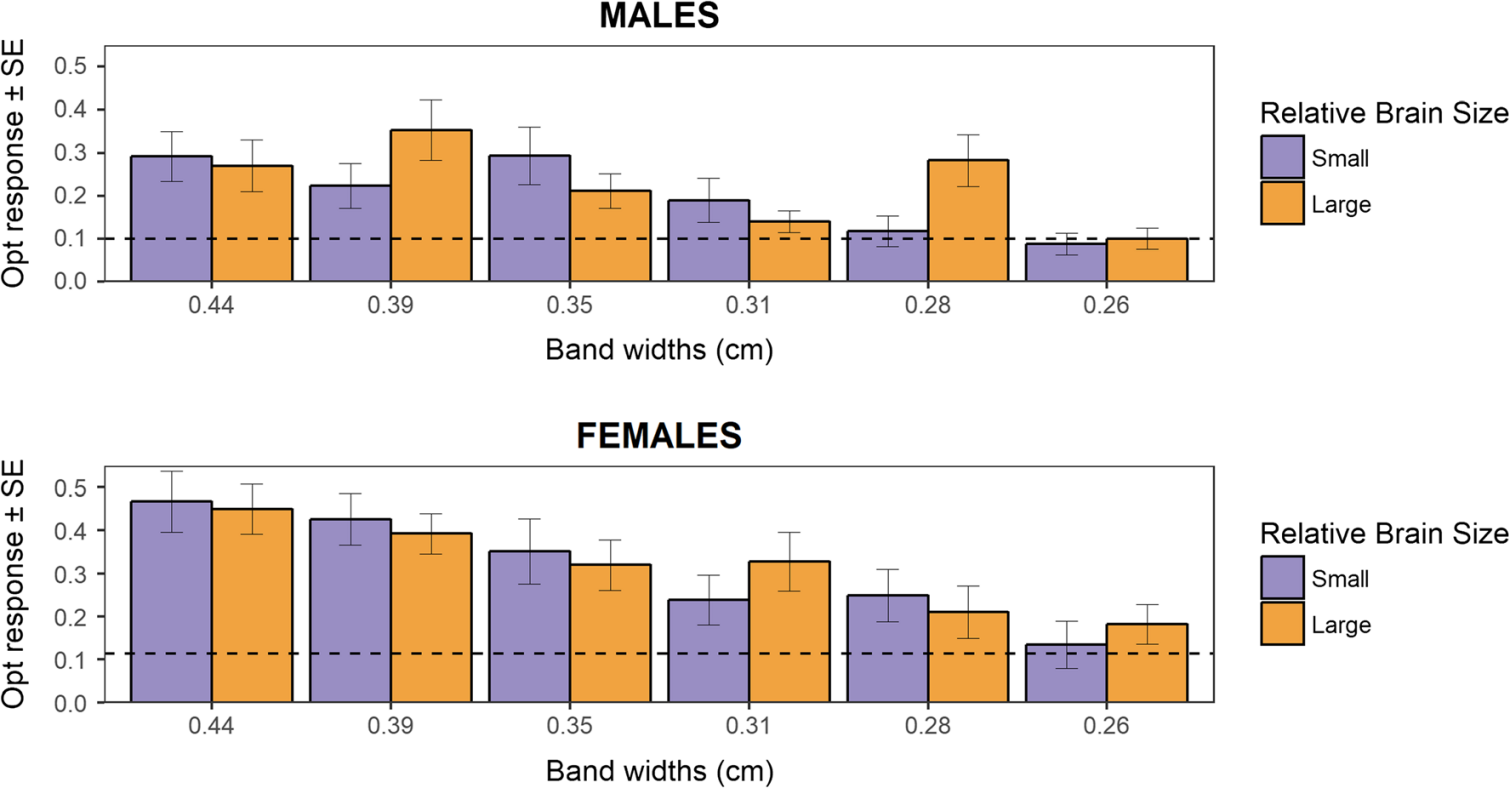
The effect of artificial selection for relative brain size on visual acuity. A LMM controlling for sex, body size, and replicate line indicated no difference in the optomotor response of large-brained and small-brained guppies towards rotational stimuli at the lower end of guppy visual acuity (*p* = 0.733). Independent analyses of the results for every bandwidth in males and females also did not show any effect of brain size selection regime on optomotor response (see Table S2). Dashed line indicates the average baseline optomotor response (circling clockwise) when presented with static images of the stimuli

Analyses combining all stimuli showed that fish spent a much larger proportion of time circling when presented with a rotational stimulus than when presented with static images of stimuli (means ± SE: estimate_rotating_ 0.27 ± 0.01, estimate_not rotating_ 0.10 ± 0.01; LMM_optomotor response_: rotation: *χ* ^2^ = 76.81, df = 1, *p* < 0.001). Analyses of optomotor response for every independent bandwidth showed similar patterns for all stimuli presented, with the exception of the thinnest stimulus (bandwidths of 0.20 cm; Table 1). The behavioral response to rotational stimuli was positively associated with body size (LMM_optomotor response_: body size: *χ* ^2^ = 4.62, df = 1, *p* = 0.036; Fig. 3). However, we found no effect of brain size selection regime on optomotor response (means ± SE: estimate_large-brained_ 0.26 ± 0.03, estimate_small-brained_ 0.25 ± 0.03; LMM_optomotor response_: brain size: *χ* ^2^ = 0.11, df = 1, *p* = 0.733; Fig. 4). Likewise, independent analyses of females and males in their optomotor response revealed no significant difference in visual acuity between large-brained and small-brained fish for any of the bandwidths quantified (Supplementary material 5: Table S2). Large-brained males seemed to show more pronounced optomotor response than small-brained males in one single stimulus at the lower end of guppy visual acuity. However, when applying the false discovery rate procedure, this difference is non-significant.

**Table 1:** Optomotor response towards stimuli at the lower end of guppy visual acuity

	Total optomotor response	
Bandwidth (cm)	Rotational stimulus (mean ± SE)	Static image (mean ± SE)
0.34	0.369 ± 0.027	0.112 ± 0.027
0.30	0.348 ± 0.024	0.078 ± 0.024
0.27	0.294 ± 0.025	0.102 ± 0.025
0.24	0.223 ± 0.022	0.055 ± 0.022
0.22	0.214 ± 0.025	0.138 ± 0.025
0.20	0.126 ± 0.019	0.151 ± 0.019
Average	0.262 ± 0.014	0.106 ± 0.014

Average proportion of time that fish, regardless of relative brain selection treatment (*n* = 60), spent performing an optomotor reflex movement towards rotating black and white stripes of different widths, as well as towards an static image of the stimulus

The observed effect size of relative brain size in the model was negligible (Cohen’s *d* = 0.08), indicating a strong overlap in the distribution of optomotor response in large-brained and small-brained fish. Since observed statistical power is a combination of effect size and sample size and given the negligible effect sizes in our model, the observed power of our model was equally negligible (Supplementary material 6: Fig. S4). This means that average optomotor responses quantified in our test are almost the same for large-brained and small-brained guppies. Power analysis simulations further confirmed that a large increase in sample size would not have resulted in an increase in the power to detect significant differences between our treatments (Supplementary material 6: Fig. S4). These results suggest that there is no biologically relevant effect on visual acuity resulting from strong selection on relative brain size in guppies.

**Fig. 5:**
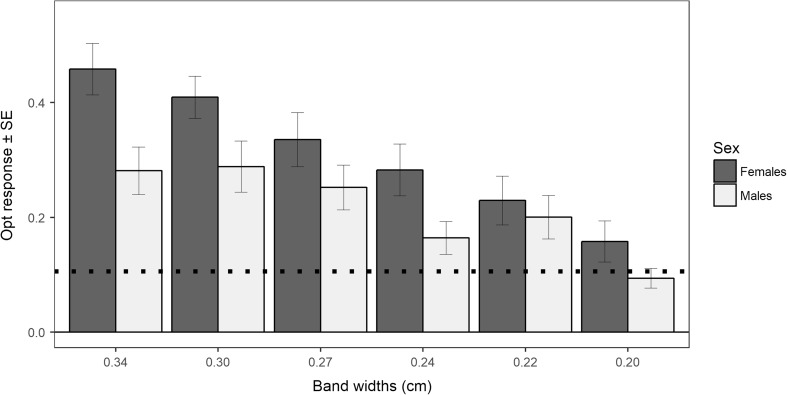
Sex differences in visual acuity. Female guppies regardless of relative brain size selection (*n* = 30) presented a higher optomotor response than male guppies (*n* = 30) towards all rotational stimuli at the lower end of guppy visual acuity (average optomor response ± SE: females 0.31 ± 0.02; males 0.21 ± 0.02; p = 0.004). However, no significant difference in optomotor response between the sexes was observed in a LMM which controlled for the effect of body size and brain size selection treatment (*p* = 0.974). Dashed line indicates the average baseline optomotor response (circling clockwise) when presented with static images of the stimuli

Analyses of sex differences on visual acuity revealed that although females presented higher average optomotor response than males for all stimuli (means ± SE: estimate_females_ (*n* = 30) 0.31 ± 0.02, estimate_males_ (*n* = 30) 0.21 ± 0.02; LMM_optomotor response_: sex: χ2 = 8.05, df = 1, p = 0.004;  Fig. 5), this difference was likely driven by their larger body size since we found no effect of sex on optomotor response when controlling for body size (LMM_optomotor response_: sex: *χ* ^2^ = 0.01, df = 1, *p* = 0.974).

## Discussion

Behavioral analyses of the optomotor response revealed that larger individuals have better visual acuity than smaller individuals in this species. But, selection for relative brain size did not result in visual acuity differences between the large-brained and small-brained guppies, which seems surprising as we did find an average 6.35% increase of eye size with increased relative brain size. This observed level of increase using a coarse measure of the visual system is in concordance with the concerted increase shown for all major brain regions in relative brain size-selected guppies (Kotrschal et al. 2017), further stressing the consideration of the visual system as an integrated part of the brain.

The strong relationship between body size and visual acuity that we find is in agreement with previous research performed in insects. Across foraging bee species (*Apoidea*), unlike ecological factors, body size was found to be positively correlated with the number of ommatidia per eye (Jander and Jander 2002). The ommatidia are clusters of photoreceptor cells in the insect eyes, and their abundance is the determining factor for visual abilities in this taxa (Land and Nilsson 2012). Several studies concur with this finding, as acuity and sensitivity positively correlate with body size in different species of butterflies (Rutowski 2000; Rutowski et al. 2009). Moreover, research across teleost fishes has established a concerted increase of the ocular components of the visual system with body size (Powers and Raymond 1990). Our results are in line with this concerted increase, as females, which were on average 35% larger than males, had 37.3% larger eyes in the sample used for this study. Given the body size sexual dimorphism in this species (Houde 1997), our finding of a positive correlation between body size and visual acuity indicates that females possess better visual acuity than males (see Fig. 5). Such differences in body size and eye size may contribute to increase the number of photoreceptors in the retina of these fish, a factor that has been argued to contribute to a higher visual acuity in larger fish species (reviewed in Douglas and Hawryshyn 1990). Given the strong effect of body size in our results, we think that it is unlikely that the prominent ecological differences between the sexes in guppies (Magurran 2005) influenced the observed differences in visual acuity. Yet, it is possible that ecological factors play a more prominent role in other visual capabilities of male and female guppies. For instance, the potential role for sexual selection in shaping the previously shown differences in the expression of color vision-related genes between males and female guppies (Corral-López et al. 2017). Regardless of the proximate cause of these observed visual acuity differences, it is possible that they contribute to the observed difference in performance between males and females in visual discriminating tasks, where females often outperform males (Kotrschal et al. 2013; van der Bijl et al. 2015; Lucon-Xiccato et al. 2016).

Why did we not detect any effect of eye size on differences in visual acuity between large-brained and small-brained guppies? Large-brained individuals present both larger absolute values and larger relative values of eye area. Unlike for the larger sex differences, these differences did not translate into performance differences between large-brained and small-brained fish in the visual acuity assays. Previous studies have showed a linear correlation between visual acuity and eye size across ray-finned fishes (Caves et al. 2017). However, it seems plausible that changes in the eye morphology influence visual acuity only after a certain difference threshold is exceeded. Large-brained fish had 6.7% larger eyes than small-brained fish, far from the observed 37.3% difference between males and females. Indeed, previous morphological studies in goldfish suggest a non-linear relationship between the increase in eye size and the decrease of the density of eye photoreceptors (Hester 1968). Differences in body size might help to exceed such threshold in eye size also, but there is no body size difference between large-brained and small-brained individuals, either between the here tested fish, or in previous assays that used a much larger number of fish (Kotrschal et al. 2013). Alternatively, differences between our brain size selection lines in eye morphological constraints and in juvenile growth (Kotrschal et al. 2015a) may trigger changes in the composition of photoreceptor cells and retinal ganglion cells of the eye during ontogeny (Kotrschal et al. 1990). In addition, most of the retina provides low visual acuity information to the central nervous system (Fernández-Juricic 2012), and only a small area, the *retinal specialization area* (Meyer 1977), processes high visual acuity information. Potentially, the difference we observed in eye size might not translate into photoreceptor and/or ganglion cell number differences in this specific area.

At the macroevolutionary level, ecological factors influencing the light regime, diel patterns, or feeding ecotype seem to be important in shaping the visual system of the species (Huber et al. 1997; Hutcheon et al. 2002; Veilleux and Kirk 2014; Caves et al. 2017). In particular, studies on the visual system of fish found that adaptations to different light intensities are connected to a higher growth of the outer segments of cone and rod photoreceptors within the retina (Wagner 1990). Given our findings on eye size differences between the brain size-selected lines, we speculate that large-brained individuals might have a higher potential to adapt to environments with different light conditions. Yet, despite the observed morphological differences in the visual organ, visual capabilities between large-brained and small-brained individuals are very consistent in our tests where light regime is kept constant during ontogeny. It is unclear what effect selection for larger brains may have on neural wiring and cell composition within the brain of male and female guppies. Yet, our findings in this study suggest that the overall concerted increase of all major brain regions, including the visual region, accounts for the observed behavioral differences previously shown in experimental tests where visual ability could play an important role.

We conclude that the positive relationship we found between body size and visual acuity stresses the importance of accounting for pre-existing perceptual biases in behavioral studies (Ryan and Cummings 2013). As such, we think that integrating the role of sensory mechanisms during experimental manipulations performed in animal behavior studies needs to be taken seriously. In a recent article, Jordan and Ryan (2015) advocated for a correct use of the perceptual space of organisms in the rapidly developing field of adaptive behavioral landscapes. We fully support this view and suggest a widespread extension of these practices to experimental behavioral studies.

## Electronic supplementary material

ESM 1 (PDF 472 kb)

## References

[CR1] Anstis S, Hutahajan P, Cavanagh P (1998). Optomotor test for wavelength sensitivity in guppyfish (*Poecilia reticulata*). Vis Res.

[CR2] Benjamini Y, Hochberg Y (1995). Controlling the false discovery rate: a practical and powerful approach to multiple testing. J Roy Stat Soc B Met.

[CR3] Brandstätter R, Kotrschal K (1990). Brain growth patterns in four European cyprinid fish species (Cyprinidae Teleostei): roach (*Rutilus rutilus*), bream (*Abramis brama*), common carp (*Cyprinus carpio*), and sabre carp (*Pelecus cultratus*). Brain Behav Evol.

[CR4] Caves E, Sutton TT, Johnsen S (2017). Visual acuity in ray-finned fishes correlates with eye size and habitat. J Exp Biol.

[CR5] Clark DT (1981) Visual responses in developing zebrafish (*Brachydanio rerio*). PhD thesis, University of Oregon

[CR6] Collin SP, Archer SN, Djamgoz MBA, Loew ER, Partridge JC, Vallerga S (1999). Behavioural ecology and retinal cell topography. Adaptive mechanisms in the ecology of vision.

[CR7] Corral-López A, Bloch NI, Kotrschal A, van der Bijl W, Buechel SD, Mank JE, Kolm N (2017). Female brain size affects the assessment of male attractiveness during mate choice. Sci Adv.

[CR8] Douglas RH, Hawryshyn CW, Douglas RH, Djamgoz MBH (1990). Behavioural studies of fish vision: an analysis of visual capabilities. The visual system of fish.

[CR9] Fernández-Juricic E (2012). Sensory basis of vigilance behavior in birds: synthesis and future prospects. Behav Process.

[CR10] Friard O, Gamba M (2016). BORIS: a free versatile open-source event-logging software for video/audio coding and live observations. Methods Ecol Evol.

[CR11] Green P, MacLeod CJ (2016). SIMR: an R package for power analysis of generalized linear mixed models by simulation. Methods Ecol Evol.

[CR12] Hall MI, Heesy CP (2011). Eye size, flight speed and Leuckart’s law in birds. J Zool.

[CR13] Haug MF, Biehlmaier O, Mueller KP, Neuhauss SC (2010). Visual acuity in larval zebrafish: behavior and histology. Front Zool.

[CR14] Heesy CP, Hall MI (2010). The nocturnal bottleneck and the evolution of mammalian vision. Brain Behav Evol.

[CR15] Hester FJ (1968). Visual contrast thresholds of the goldfish (*Carassius auratus*). Vis Res.

[CR16] Houde AE (1997). Sex, color and mate choice in guppies.

[CR17] Huber R, van Staaden MJ, Kaufman LS, Liem KF (1997). Microhabitat use trophic patterns and the evolution of brain structure in African cichlids. Brain Behav Evol.

[CR18] Hughes A, Autrum H (1977). The topography of vision in mammals of contrasting lifestyle: comparative optics and retinal organisation. Handbook of sensory physiology.

[CR19] Hutcheon JM, Kirsch JA, Garland T (2002). A comparative analysis of brain size in relation to foraging ecology and phylogeny in the *Chiroptera*. Brain Behav Evol.

[CR20] Iwaniuk A, Kaas JH (2016). Functional correlates of brain and brain region sizes in nonmammalian vertebrates. Evolution of nervous systems.

[CR21] Jander U, Jander R (2002). Allometry and resolution of bee eyes (*Apoidea*). Arthropod Struct Dev.

[CR22] Jordan LA, Ryan MJ (2015). The sensory ecology of adaptive landscapes. Biol Lett.

[CR23] Kotrschal K, Adam H, Brandstätter R, Junger H, Zaunreiter M, Goldschmid A (1990). Larval size constraints determine directional ontogenetic shifts in the visual system of teleosts. J Zool Syst Evol Res.

[CR24] Kotrschal A, Corral-López A, Amcoff M, Kolm N (2015). A larger brain confers a benefit in a spatial mate search learning task in male guppies. Behav Ecol.

[CR25] Kotrschal A, Corral-López A, Szidat S, Kolm N (2015). The effect of brain size evolution on feeding propensity digestive efficiency and juvenile growth. Evolution.

[CR26] Kotrschal A, Kolm N, Penn DJ (2016). Selection for brain size impairs innate but not adaptive immune responses. Proc R Soc B.

[CR27] Kotrschal A, Rogell B, Bundsen A, Svensson B, Zajitschek S, Brännström I, Immler S, Maklakov AA, Kolm N (2013). Artificial selection on relative brain size in the guppy reveals costs and benefits of evolving a larger brain. Curr Biol.

[CR28] Kotrschal A, Zeng HL, van der Bijl W, Oehman-Mägi C, Pelckmans K, Kolm N (2017) Evolution of brain region volumes during artificial selection for relative brain size. Evolution (published online). 10.1111/evo.13373 10.1111/evo.13373 28986929

[CR29] Kuzawa CW, Chugani HT, Grossman LI, Lipovich L, Muzik O, Hof PR, Wildman DE, Sherwood CC, Leonard WR, Lange N (2014). Metabolic costs and evolutionary implications of human brain development. P Natl Acad Sci USA.

[CR30] Land MF, Nilsson DE (2012). Animal eyes.

[CR31] Lucon-Xiccato T, Dadda M, Bisazza A (2016). Sex differences in discrimination of shoal size in the guppy (*Poecilia reticulata*). Ethology.

[CR32] Magurran AE (2005). Evolutionary ecology: the Trinidadian guppy.

[CR33] Meyer DBC, Crescitelli F (1977). The avian eye and its adaptations. The visual system of vertebrates. Handbook of sensory physiology.

[CR34] Nakagawa S, Cuthill IC (2007). Effect size confidence interval and statistical significance: a practical guide for biologists. Biol Rev.

[CR35] Neave DA (1984). The development of visual acuity in larval plaice (*Pleuronectes platessa* L.) and turbot (*Scophthalmusmaximus* L.). J Exp Mar Biol Ecol.

[CR36] Niven JE (2016). Neuronal energy consumption: biophysics efficiency and evolution. Curr Opin Neurobiol.

[CR37] Niven JE, Laughlin SB (2008). Energy limitation as a selective pressure on the evolution of sensory systems. J Exp Biol.

[CR38] Powers MK, Raymond PA, Douglas RH, Djamgoz MBH (1990). Development of the visual system. The visual system of fish.

[CR39] R Core Team (2015). R: a language and environment for statistical computing.

[CR40] Roderick TH, Wimer RE, Wimer CC, Schwartzkroin PA (1973). Genetic and phenotypic variation in weight of brain and spinal cord between inbred strains of mice. Brain Res.

[CR41] Ryan MJ, Cummings ME (2013). Perceptual biases and mate choice. Annu Rev Ecol Evol Syst.

[CR42] Rutowski RL (2000). Variation of eye size in butterflies: inter-and intraspecific patterns. J Zool.

[CR43] Rutowski RL, Gislén L, Warrant EJ (2009). Visual acuity and sensitivity increase allometrically with body size in butterflies. Arthropod Struct Dev.

[CR44] van der Bijl W, Thyselius M, Kotrschal A, Kolm N (2015). Brain size affects the behavioural response to predators in female guppies (*Poecilia reticulata*). Proc R Soc B.

[CR45] von der Emde G, Warrant E (2016). The ecology of animal senses.

[CR46] Schneider CA, Rasband WS, Eliceiri KW (2012). NIH image to ImageJ: 25 years of image analysis. Nat Methods.

[CR47] Veilleux CC, Kirk EC (2014). Visual acuity in mammals: effects of eye size and ecology. Brain Behav Evol.

[CR48] Wagner HJ, Douglas RH, Djamgoz MBH (1990). Retinal structure of fishes. The visual system of fish.

[CR49] Wylie DR, Gutiérrez-Ibáñez C, Iwaniuk AN (2015). Integrating brain, behavior and phylogeny to understand the evolution of sensory systems in birds. Front Neurosci.

[CR50] Yopak KE, Lisney TJ (2012). Allometric scaling of the optic tectum in cartilaginous fishes. Brain Behav Evol.

