# Supplementary material for: On the role of body size, brain size, and eye size in visual acuity
Source: Behav Ecol Sociobiol. 2017 Nov 29;71(12):179. doi: 10.1007/s00265-017-2408-z (PMC5705735; doi:10.1007/s00265-017-2408-z)
Supplement: Supplementary file 1 — (PDF 472 kb) [file 265_2017_2408_MOESM1_ESM.pdf]

Electronic supplementary material for:

Behavioral Ecology and Sociobiology

On the role of body size, brain size and eye size in visual acuity

Alberto Corral-López[1]*, Maddi Garate-Olaizola[1], Severine D. Buechel[1], Niclas Kolm[1],

Alexander Kotrschal[1]

1 Department of Zoology/Ethology, Stockholm University, Svante Arrhenius väg 18B. SE-10691, Stockholm, Sweden.

* corresponding author: alberto.corral@zoologi.su.se

Supplementary information 1

In order to assess the effect of the rotation speed of the stimuli on the optomotor response, we measured the optomotor response of all fish to two different stimuli (band width of 0.48 cm and 0.30 cm) at 4 different speeds. We used a LMM with the optomotor response as the dependent variable, stimulus speed and band width as fixed effects, and fish id as a random factor to account for the repeated measurements taken to each individual. We found a significant effect of band width in the optomotor response of the fish (estimate 0.48 cm: 0.40 ± 0.03; estimate 0.30 cm: 0.25 ± 0.03; $LMM_{\text{optomotor response:}}$ width: $\chi 2$ (1) = 85.94, $p < 0.001$; Fig. S1). However, we found no clear effect of the rotation speed of the stimulus in the optomotor response (estimate slow: 0.31 ± 0.03; estimate med1: 0.31 ± 0.03; estimate med2: 0.35 ± 0.03; estimate fast: 0.31 ± 0.03; $LMM_{\text{optomotor response:}}$ speed: $\chi 2$ (3) = 2.17, $p = 0.090$; Fig. S1).

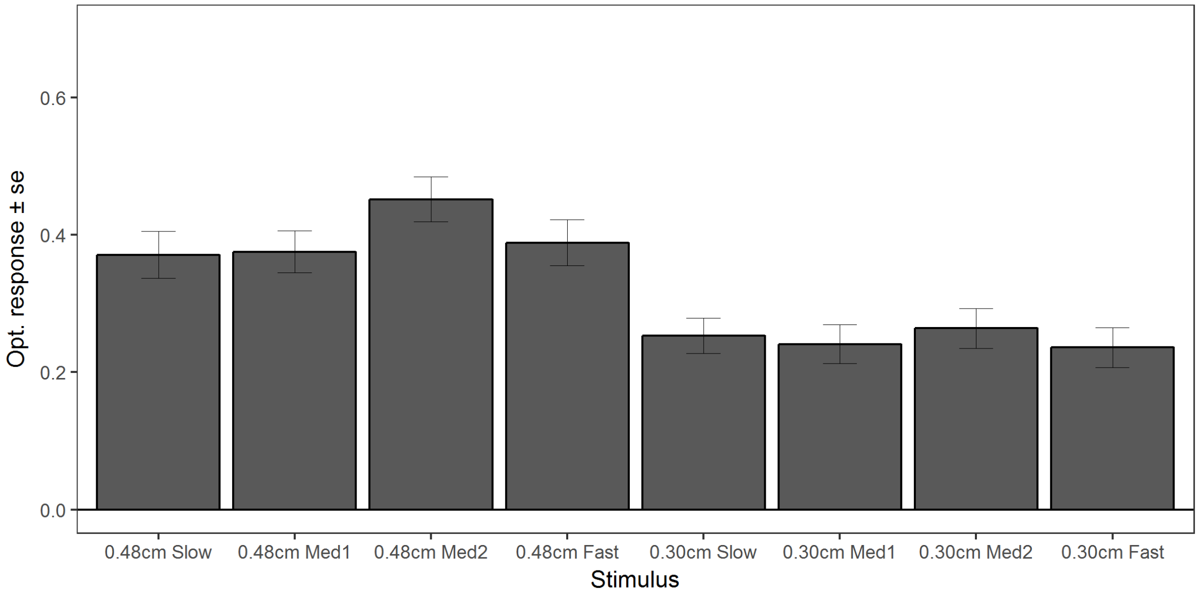

Fig. S1 Effect of rotation speed on the optomotor response towards black and white striped rotational stimulus. Optomotor response of female and male guppies artificially selected for relative brain size (n = 60) measured at two different stripe widths showed no clear differences in response to variation in the speed at which the stimuli were rotating (p = 0.090). Optomotor response significantly decreased with decreasing stripe width (p < 0.001)

Supplementary information 2

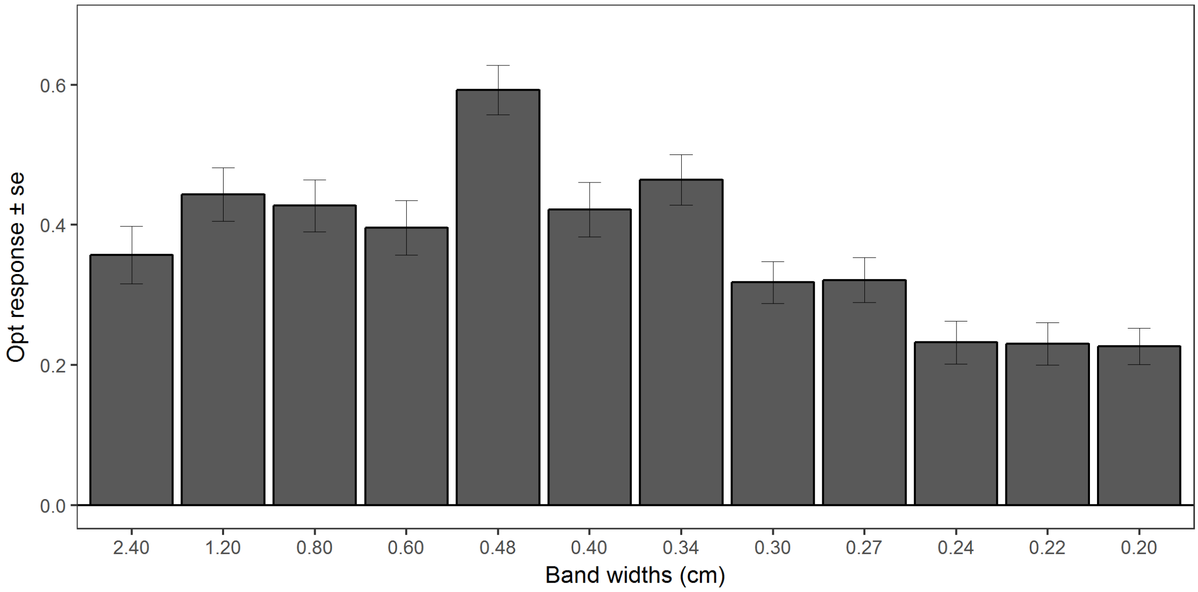

Fig. S2 Pilot test of the optomotor response in a large range of band widths. The optomotor response of male (n = 30) and female (n = 30) guppies artificially selected for relative brain size was recorded when exposed, in a random order, to a 60 seconds rotating stimuli of black and white stripes in the range between 2.40 cm to 0.20 cm. Visual inspection of the measurements suggested a lower reflex response towards the stimuli in the widths ranged between 0.20 and 0.30 cm

Supplementary information 3

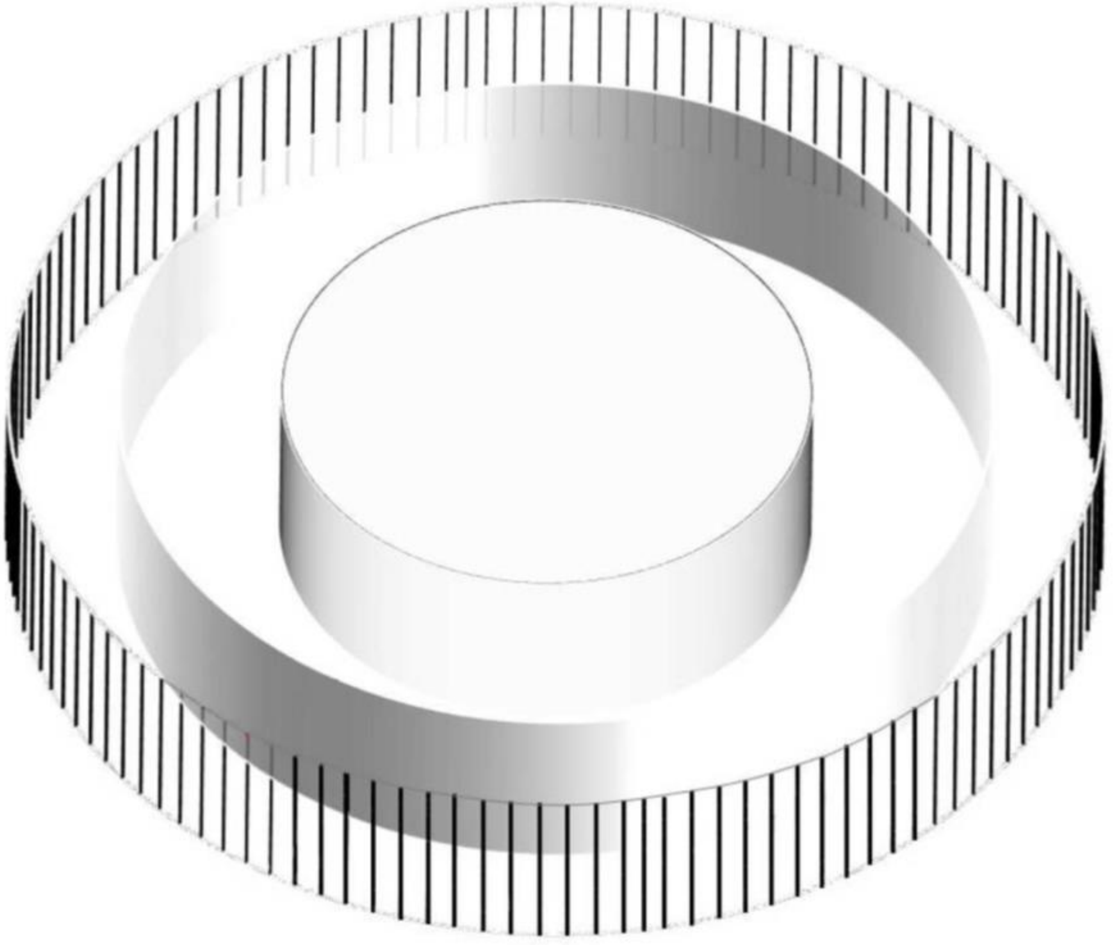

Fig. S3 Schematics of experimental setup used in visual acuity tests. Three-dimensional sketch of the experimental set-up used to quantify the optomotor response towards rotational stimuli in large- and small-brained guppies. Black and white bands of different widths were projected in the external wall of a white tank with 50 cm of outer diameter and 25 cm of inner diameter. During tests, we placed a transparent Perspex ring of 40 cm diameter to only allow fish to swim with a minimum distance of 10 cm of the stimulus projection

Supplementary information 4

To study the effect of sex and relative brain size in visual acuity, we used a LMM with optomotor response as the dependent variable. We used sex and brain size selection regime as fixed effects, and fish body size as a covariate. Full models included all interactions between fixed effects and the covariate. In addition, models included a random intercept for each replicate selection line and a random slope for brain size within each replicate. Stepwise model selection based on Akkaike Information Criterion indicated a better fit of the model without any interaction between fixed effects and the covariate.

R syntax of every model:

Model1 <- lmer(optresponse_r ~ bs*sex*bodysize + (1|bs:rep))

Model2 <- lmer(optresponse_r ~ bs + sex + bodysize + bs:sex + bs:bodysize + sex:bodysize + (1|bs:rep))

Model3 <- lmer(optresponse_r ~ bs + sex + bodysize + bs:sex + bs:bodysize + (1|bs:rep))

Model4 <- lmer(optresponse_r ~ bs + sex + bodysize + bs:bodysize + sex:bodysize + (1|bs:rep))

Model5 <- lmer(optresponse_r ~ bs + sex + bodysize + bs:sex + sex:bodysize + (1|bs:rep))

Model6 <- lmer(optresponse_r ~ bs + sex*bodysize + (1|bs:rep))

Model7 <- lmer(optresponse_r ~ sex + bs*bodysize + (1|bs:rep))

Model8 <- lmer(optresponse_r ~ bs * sex + bodysize + (1|bs:rep))

Model9 <- lmer(optresponse_r ~ bs + sex + bodysize + (1|bs:rep))

Model results:

Table S1 Results from a LMM models used in the stepwise model selection process.
Model 9 presented the best fit indicated by its lower AIC value

Model 1

Response = Opt. Response AIC = -31.207

	NumDF	DenDF	F	p-value
brain size	1	51.950	0.018	0.890
sex	1	50.750	0.014	0.900
body size	1	51.990	4.555	0.037*
brain size:sex	1	50.750	0.432	0.513
brain size:body size	1	51.990	0.073	0.787
sex:body size	1	50.790	0.018	0.893
brain size:body size:sex	1	50.790	0.505	0.480

Model 2

Response = Opt. Response AIC = -32.792

	NumDF	DenDF	F	p-value
brain size	1	52.902	0.003	0.955
sex	1	51.661	0.008	0.927
body size	1	52.474	4.222	0.044*
brain size:sex	1	52.638	0.080	0.779
brain size:body size	1	52.997	0.010	0.917
sex:body size	1	51.870	0.007	0.931

Model 3

Response = Opt. Response AIC = -36.281

	NumDF	DenDF	F	p-value
brain size	1	53.936	0.001	0.955
sex	1	52.833	0.000	0.927
body size	1	53.731	4.469	0.039*
brain size:sex	1	52.833	0.072	0.788
brain size:body size	1	53.731	0.007	0.931

Model 4

Response = Opt. Response AIC = -37.162

	NumDF	DenDF	F	p-value
brain size	1	53.579	0.083	0.774
sex	1	53.473	0.001	0.970
body size	1	53.556	4.297	0.043*
brain size:body size	1	52.157	0.045	0.832
sex:body size	1	53.570	0.001	0.973

Model 5

Response = Opt. Response AIC = -36.331

	NumDF	DenDF	F	p-value
brain size	1	3.753	0.243	0.649

sex	1	53.674	0.005	0.943
body size	1	53.510	4.309	0.042*
brain size:sex	1	50.037	0.113	0.737
sex:body size	1	53.738	0.004	0.946

Model 6

Response = Opt. Response AIC = -41.723

	NumDF	DenDF	F	p-value
brain size	1	3.757	0.243	0.649
sex	1	54.672	0.003	0.954
body size	1	54.516	4.344	0.041*
sex:body size	1	54.737	0.002	0.957

Model 7

Response = Opt. Response AIC = -40.702

	NumDF	DenDF	F	p-value
brain size	1	54.420	0.086	0.769
sex	1	53.835	0.000	0.982
body size	1	54.730	4.587	0.036*
brain size:body size	1	52.842	0.047	0.828

Model 8

Response = Opt. Response AIC = -39.871

	NumDF	DenDF	F	p-value
brain size	1	3.873	0.250	0.643
sex	1	53.769	0.000	0.978
body size	1	54.667	4.571	0.036*
brain size:sex	1	50.957	0.113	0.737

Model 9

Response = Opt. Response AIC = -45.282

	NumDF	DenDF	F	p-value
brain size	1	3.874	0.250	0.644
sex	1	54.773	0.001	0.974
body size	1	55.679	4.618	0.036*

Supplementary information 5

Table S2 Analyses of optomotor response for independent band width measurements. We found no significant differences between large-brained and small-brained fish in the optomotor response measured for any rotational stimulus used. We used a LMM with the optomotor response as the dependent variable, brain size selection regime as fixed effects, and body size as a covariate. All models included a random intercept for each replicate selection line and a random slope for brain size selection regime within each replicate. Independent analyses were done for males and females. Due to multiple testing, we used a false discovery rate procedure (Benjamini and Hochberg 1995), but the only p-value significant (0.026) under the 0.05 significance level did not satisfy the constraint (0.026 > p = 0.05*1 / 6 = 0.008)

FEMALES

	Total optomotor response					
Band thickness (cm)	Small-brained (Mean ± SE)	Large-brained (Mean ± SE)	Num DF	Den DF	F	p-value
0.34	0.469 ± 0.080	0.447 ± 0.080	1	3.508	0.034	0.862
0.30	0.424 ± 0.073	0.395 ± 0.072	1	3.921	0.078	0.793
0.27	0.349 ± 0.082	0.321 ± 0.082	1	3.497	0.060	0.819
0.24	0.238 ± 0.065	0.327 ± 0.065	1	3.690	0.922	0.345
0.22	0.242 ± 0.064	0.216 ± 0.065	1	3.819	0.086	0.783
0.20	0.132 ± 0.056	0.184 ± 0.056	1	3.948	0.433	0.547

MALES

	Total optomotor response					
Band thickness (cm)	Small-brained (Mean ± SE)	Large-brained (Mean ± SE)	Num DF	Den DF	F	p-value
0.34	0.291 ± 0.060	0.271 ± 0.060	1	3.770	0.055	0.816
0.30	0.219 ± 0.060	0.357 ± 0.059	1	3.681	2.680	0.113
0.27	0.290 ± 0.054	0.214 ± 0.054	1	4.003	0.967	0.380
0.24	0.187 ± 0.041	0.140 ± 0.040	1	3.764	0.665	0.421
0.22	0.117 ± 0.050	0.283 ± 0.050	1	3.588	5.505	0.026
0.20	0.086 ± 0.024	0.101 ± 0.024	1	3.724	0.170	0.683

Supplementary information 6

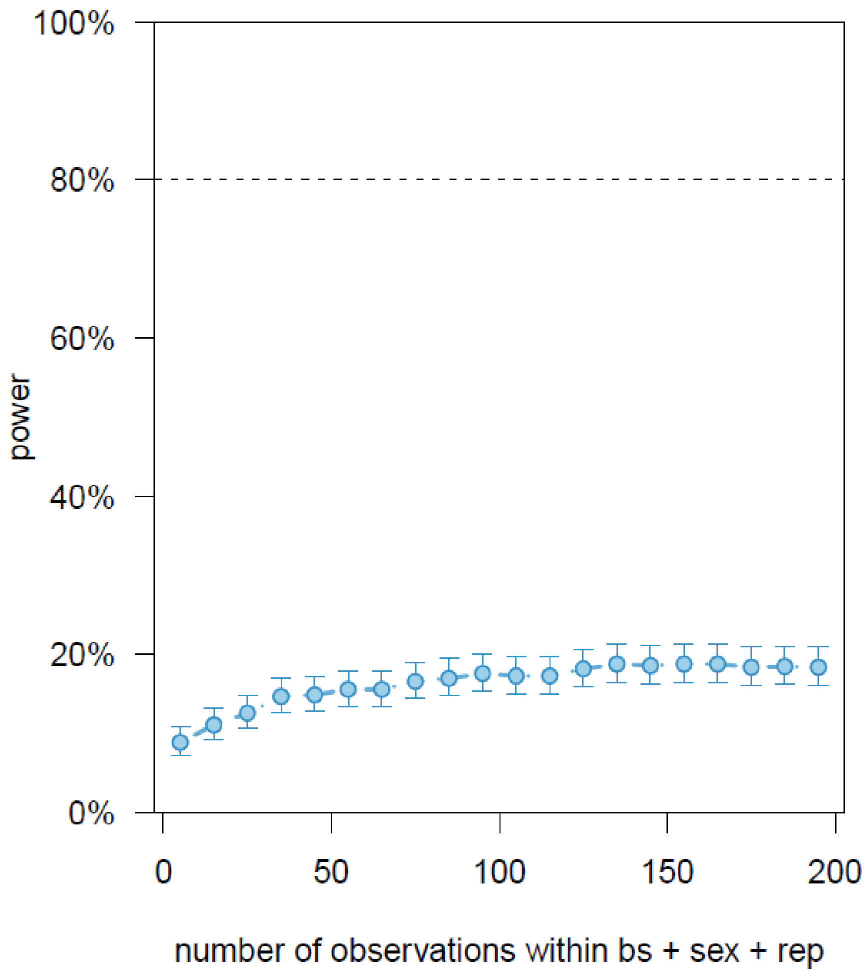

Fig. S4 Observed power analysis simulations on the effect of relative brain size on visual acuity of guppies. Based on the sample size used (n = 60) and the effect size obtained in a LMM testing the effect of relative brain size in the optomotor response of guppies (Cohen's D = 0.08), power simulations indicated that even a large increase in sample size would not result in an increase of the observed power of the test over 20%. This indicates that there is no biologically relevant effect on visual acuity resulting from a rapid selection on relative brain size in guppies. X- axis show the increase in sample size of individuals that belong to the same sex, brain size selection regime and replicate line necessary to increase a given observed power
